# Assessing the Validity and Acceptability of an Adult Quality of Life Questionnaire, the EORTC QLQ‐C30, for Adolescents With Cancer

**DOI:** 10.1002/cam4.71952

**Published:** 2026-05-21

**Authors:** Samantha C. Sodergren, David Riedl, Jamal Hossain, Haneen Abaza, Amal Al‐Omari, Carlota Aguilera Ordoñez, Andrea Artigas Ramiro, Chiara Besani, Jaques Van Heerden, Lisa Lyngsie Hjalgrim, Betty Illatopa, Hiroto Ishiki, Manjunath Nookala Krishnamurthy, Lucas Moreno, Rebecca Mottram, Stephanie O'Toole, Marta Pérez‐Campdepadros, Jelena Roganovic, Anna Salo, Caroline De Schepper, Mark P. Tighe, Anne‐Sophie Darlington, the EORTC Quality of Life Group

**Affiliations:** ^1^ University of Southampton Southampton UK; ^2^ University Hospital of Psychiatry II, Medical University of Innsbruck Innsbruck Austria; ^3^ Ludwig Boltzmann Institute for Rehabilitation Research Vienna Austria; ^4^ King Hussein Cancer Center Office of Scientific Affairs and Research Amman Jordan; ^5^ Vall d'Hebron University Hospital Barcelona Spain; ^6^ Hospital Niño Jesús Madrid Spain; ^7^ Children's Hospital Ireland Dublin Ireland; ^8^ Antwerp University Hospital Antwerp Belgium; ^9^ Copenhagen University Hospital – Rigshospitalet Copenhagen Denmark; ^10^ National Cancer Center Hospital Tokyo Japan; ^11^ Advanced Centre for Treatment, Research and Education in Cancer Kharghar Maharashtra India; ^12^ HomiBhabha National Institute Mumbai India; ^13^ Leeds Childrens Hospital Leeds UK; ^14^ Pediatric Cancer Centre Hospital Sant Joan de Déu Barcelona Spain; ^15^ Department of Child and Adolescent Mental Health Hospital Sant Joan de Déu Barcelona Spain; ^16^ Children's Hospital Zagreb Zagreb Croatia; ^17^ University Hospitals Dorset NHS Foundation Trust Poole UK

**Keywords:** adolescents, cancer, EORTC QLQ‐C30, patient‐reported outcomes, psychometric validation, quality of life

## Abstract

**Background:**

The EORTC QLQ‐C30 (QLQ‐C30) is the most widely used cancer‐specific patient‐reported outcome questionnaire in adult clinical trials, but its suitability for adolescents who have distinct developmental needs is unclear. Adolescents with cancer are often underrepresented in clinical trials, and efforts are underway to improve their participation by lowering the age of entry. This study evaluated the QLQ‐C30's acceptability, reliability, validity and delivery preferences in adolescents with cancer across different languages and cultural backgrounds.

**Materials and Methods:**

Adolescents aged 12–17 years, undergoing or having completed cancer treatment for curative or palliative intent, completed the QLQ‐C30 alongside the PedsQL Cancer Teen questionnaire and shared their feedback on relevance, ease of completion and preferred mode of administration (paper or electronic). Socio‐demographic and clinical data were recorded.

**Results:**

Two hundred adolescents (mean age 14.5 years, standard deviation (SD) 1.6 years; 51.5% male) representing different cultures participated. The QLQ‐C30 required a mean 12 min to complete and was described as acceptable and easy to complete. Psychometric testing confirmed the QLQ‐C30 subscale structure with acceptable‐to‐good internal consistency (Cronbach's α 0.70–0.84), as well as good convergent and discriminant validity with PedsQL subscales. Comparisons of scores according to treatment intent and performance status demonstrated some support for known‐group validity. Over half (57%) adolescents preferred paper completion of questionnaires.

**Conclusions:**

This study is the first to systematically evaluate the QLQ‐C30 in adolescents and establishes its acceptability and validity in this population, confirming it as a measure of choice for clinical trials involving adolescents and enabling robust life course‐based outcome assessment.

## 1 Introduction

Health‐related quality of life (HRQoL) plays a central role in health outcome assessment in cancer, providing valuable insight into the impact of cancer and its treatment from the patient's perspective. Patient‐reported outcomes (PROs) are increasingly incorporated into clinical trial protocols alongside survival‐ and disease‐related endpoints and are strongly endorsed by regulatory agencies [1], including the US Food and Drug Administration [2] and the European Medicines Agency [3]. In addition to playing a crucial role in the assessment of the efficacy and safety of new treatments, PROs provide valuable insight into HRQoL, which might otherwise be overlooked [4]. With careful consideration and monitoring, HRQL can guide treatment timing and co‐adjuvant medications to mitigate side effects [5]. Given this pivotal role across different contexts, it is vital that assessment tools are sensitive to the specific concerns of the target patient population [6].

The cross‐culturally valid core cancer HRQoL questionnaire of the European Organisation for Research of Cancer (EORTC), the EORTC QLQ‐C30 (QLQ‐C30) [7] is the most widely used PRO in cancer clinical trials [8, 9] and practice [10], used in 5000 studies worldwide and available in more than 120 different languages [11].

PRO assessment in paediatric oncology has been reported as falling behind adult oncology [12, 13]. Furthermore, despite the recommendation to use PROs as endpoints in clinical trials, and the published paediatric oncology guidelines to facilitate the use of PROs [14], they are not commonplace within trials involving children and adolescents. Usage of PROs is as low as 8.2% for childhood cancer clinical trials, with PROs representing primary endpoints in 0.6% of cases [12]. For adolescents and young adults (AYAs), 20% of trials included PROs in their design [15] and for older adults, this rises to over a third [15, 16]. While there is no gold‐standard PRO assessment for children and adolescents with cancer, the Paediatric Quality of Life Inventory (PedsQL) [17] is most frequently used in trials but does not lend itself to comparisons with adult populations.

The QLQ‐C30 was originally developed as an adult measure (≥ 18 years); however, it has been part of research protocols involving children, including three of the 58 paediatric trials identified by Riedl et al. [12] and two subsequent studies [18, 19]. Even when the QLQ‐C30 is reserved for adult participants, the age definition of adult can be as low as 16 years [20].

The need to formally test the QLQ‐C30 with patients younger than 18 years is important not only because it is already used in this age group without any support for its suitability but also to promote its inclusion in adolescent clinical trials. There is currently a lack of robust evidence evaluating whether the QLQ‐C30 is valid and acceptable for adolescent populations, representing a critical gap in patient‐reported outcome measurement in this age group. Adolescents and young adults with cancer are at a disadvantage in terms of their outcomes compared with children and older adults [21, 22] attributed in part to their lower rates of clinical trial enrolment [23, 24]. The ACCELERATE consortium leads efforts to lower the minimum age of inclusion in adult clinical trials to 12 years, providing more equitable access to treatment [25, 26]. Despite these efforts, the absence of validated, age‐appropriate outcome measures may limit the meaningful inclusion and assessment of adolescents within such trials. Establishing evidence for the measure's validity and utility in both adult and adolescent populations would support the adoption of a unified core instrument across age groups, thereby eliminating the need for separate age‐specific measures and facilitating a life‐course approach to assessment.

Given the reports of sub‐optimal completion of PROS in cancer clinical trials [27], it is vital to take into consideration user preferences, including questionnaire completion platforms. There has been a shift towards the delivery of PRO assessments electronically to reduce costs and enhance data accuracy and completeness [28, 29]. Electronic completion of questionnaires is likely to be favoured by younger patients who, compared to their older counterparts, are more digitally literate [30]. Nevertheless, evidence regarding adolescents' preferences and experiences with different modes of PRO completion remains limited, representing an additional gap in the literature.

The overall aim of this study is to validate the QLQ‐C30 for adolescents with cancer, aged between 12 and 17 years. The study objectives are as follows: (1) Test the psychometric properties of the QLQ‐C30 with adolescents; (2) Evaluate adolescents' experiences of completing the QLQ‐C30 including their preferences for mode of completion.

## 2 Material and Methods

### 2.1 Study Design and Setting

This mixed methods, cross‐sectional validation study was conducted across 10 countries globally.

### 2.2 Participants

Adolescents aged 12–17 years were invited to participate if they had a diagnosis of cancer, were currently receiving treatment, palliative care or had completed treatment within the past 12 months. Adolescents who were 12 months post treatment and disease‐free, defined as ‘survivors’ [31], were excluded. A purposive sampling approach was adopted ensuring good representation of participants according to geographical region within Europe as well as inclusion of non‐European countries, cancer type, treatment type and intent, age sub‐group (12–14 years and 15–17 years) and treatment/palliative care. A sample size of 200 adolescents was set following guidance for validation studies [32].

### 2.3 Questionnaires

#### 2.3.1 QLQ‐C30

The QLQ‐C30 includes 30 questions and eight multi‐item scales: five functional (physical, role, emotional, cognitive and social) and three symptoms (fatigue, pain, nausea/vomiting) as well as a global health status/quality of life scale. In addition, there are six single questions: dyspnoea, sleep disturbance, appetite loss, constipation, diarrhoea and financial difficulties. Apart from the two global health status/quality of life scale questions, which are assessed on a seven‐point Likert scale (poor to excellent), responses are rated on a four‐point Likert response scale (‘Not at all’ to ‘Very much’). The first five questions have no timeframe of reference, while for the remaining questions participants are asked to reflect upon their status within the past week. Scores are transformed on a standardised scale between 0 and 100. Higher scores indicate greater symptom severity or better functioning. The QLQ‐C30 is exclusively patient‐reported with no proxy version available.

#### 2.3.2 PedsQL Cancer Teen

The PedsQL Cancer Teen (13–18 years) [17, 33] comprises 27 questions covering the following domains: (1) pain and hurt, (2) nausea, (3) procedural anxiety, (4) treatment anxiety, (5) worry, (6) cognitive problems, (7) perceived physical appearance and (8) communication. Questions are scored using a 5‐point Likert scale (‘Never’ to ‘Almost always’) and the recall period is 1 month. Scores are linearly transformed to a 0–100 scale, where higher scores indicate less problems and better HRQoL. The teen version of the questionnaire is designed for adolescents aged 13–18 years. Correspondence with the authors of the measure confirmed the suitability of using the teen version rather than the child version for participants aged 12 years.

#### 2.3.3 Socio‐Demographic and Clinical Questions

Socio‐demographic characteristics of adolescents were self‐recorded including sex, age, ethnicity, current education status (at home, hospital, part‐/full‐time or no education) and number and age of siblings. Clinical details extracted from medical records included date of diagnosis, cancer site, treatment type and intent (curative or palliative) and co‐morbidities. Performance status using the Eastern Cooperative Oncology Group (ECOG) scale [34] was self‐reported.

### 2.4 Procedure

Eligible adolescents were invited to participate in the study by a member of their clinical or research team during their visit to the hospital. Data collection took place face to face or remotely over the telephone or video call via Microsoft Teams. Participants were given the questionnaires to read through at the time of receiving their participant information sheet and consent (or assent) form. For those who completed the questionnaires over the telephone or video call, responses were written down by the researcher.

Adolescents were asked to complete the QLQ‐C30 first while also sharing comments in a cognitive, ‘think‐aloud’ [35] interview. Follow‐up questions asked whether any parts of the questionnaire were confusing, upsetting or irrelevant. Recommendations for new or re‐worded questions were invited. The time taken to complete the questionnaire and assistance required were also recorded. Adolescents then completed the 27‐item PedsQL Cancer Teen [17, 33] and were asked to share what they preferred or disliked about each questionnaire and which one provided the most complete picture of their experiences. Finally, preferences for mode of completion, online (via phone, tablet or computer) or on paper, were recorded.

### 2.5 Data Analysis

#### 2.5.1 Sample Characteristics

Participants' background characteristics were analysed descriptively. All statistical analyses were conducted using R version 4.3.3 [36] (R Core Team (2024)).

#### 2.5.2 Acceptability of the QLQ‐C30

Time taken to complete the questionnaire and experiences of answering the questions were analysed descriptively. The number of participants reporting that a question was confusing or difficult to answer, upsetting, irrelevant or in need of re‐wording was calculated and acceptability supported when questions were critiqued by less than 25% participants and ≥ 95% completion indicated acceptable compliance [37].

#### 2.5.3 Content Validity

Qualitative responses regarding item relevance, clarity, and omissions were analysed using a qualitative descriptive approach. Free‐text comments were transcribed verbatim from interview notes. Data were coded inductively and deductively according to questionnaire content.

Item response distributions were evaluated according to the EORTC QLG module development guidelines [37] where cut‐off points are specified to aid decision rules for the selection or retention of questions for new or adapted questionnaires. According to these guidelines, questions were regarded as relevant to adolescents if the mean scores (from ‘not at all’ 1‐ ‘very much’ 4) > 1.5 and a prevalence ratio (incidence of ‘A little’, ‘Quite a bit’ and ‘Very much’ responses (scores of 2, 3, 4) divided by the number of respondents) > 30%.

#### 2.5.4 Construct Validity

To test whether the factor structure of the QLQ‐C30 remains intact when completed by adolescents, confirmatory factor analysis (CFA) appropriate for ordinal categorical data was performed [38]. The overall fit of the factor structure was assessed by fit indices including the comparative fit index, Tucker Lewis Index and the root mean square error of approximation (RMSEA). Acceptable goodness of fit was defined as RMSEA values of < 0.06–0.08 and CFI/TLI values > 0.90 [39, 40]. The strength of the relationship between questions and the factors (subscales) was inspected with factor loadings ≥ 0.60 regarded as acceptable.

#### 2.5.5 Reliability Analysis

Internal consistency of questions within the multi‐item scales was evaluated using Cronbach's alpha; values between 0.70 and 0.79 were considered acceptable, 0.80 and 0.89 good and ≥ 0.90 excellent reliability [41].

#### 2.5.6 Convergent and Discriminant Validity

To assess convergent and discriminant validity, Spearman correlation coefficients were computed between conceptually similar and dissimilar subscales of the QLQ‐C30 and the PedsQL. Spearman correlation coefficients were computed as subscale scores showed non‐normal distributions and ordinal properties. Convergent validity was supported if subscales measuring similar constructs across instruments, such as pain, nausea, cognitive functioning, social functioning (with PedsQL communication) and emotional functioning (worry, procedural anxiety, treatment anxiety and physical appearance) were moderately to strongly correlated. Discriminant validity was indicated when correlations between unrelated subscales, such as QLQ‐C30 pain and nausea subscales and PedsQL physical appearance subscale and QLQ‐C30 cognitive functioning with PedsQL pain and hurt, were low, suggesting the measurement of distinct constructs. Spearman correlation coefficients were interpreted as follows: < 0.30 = low (weak), 0.30–0.39 = low–moderate, 0.40–0.49 = moderate and ≥ 0.50 = moderate–strong/strong. Correlations ≥ 0.50 were considered indicative of convergent validity, whereas correlations < 0.30 suggested discriminant validity; intermediate correlations were interpreted as reflecting partial or conceptual relatedness [42]. Only participants with complete data across matched subscale scores were included in the correlation analyses, with subscale scores computed using the ≥ 50% item rule as per the scoring guidelines.

#### 2.5.7 Known‐Group Validity

To assess whether the QLQ‐C30 can distinguish between groups of adolescents based on clinical status, independent samples were compared using Wilcoxon rank‐sum tests, as several subscale scores were not normally distributed and group sizes were unequal [43]. Specifically, participants were distinguished according to their performance status and treatment intent. Poorer quality of life scores (lower functioning and higher symptom scores) were hypothesised for adolescents with poorer performance status and those receiving palliative care. Effect sizes (*r*) were interpreted using established guidelines [42], where values of 0.10 indicate small effects, 0.30 moderate effects and 0.50 large effects.

#### 2.5.8 Preferences

The number of adolescents preferring one questionnaire or mode of delivery over another was calculated and reasons were analysed descriptively.

### 2.6 Handling of Missing Data

Missing data were handled according to the EORTC and PedsQL scoring manuals [44] whereby if at least half the number of questions within a scale were completed, the mean of these responses was imputed; otherwise, the scale was regarded as missing.

### 2.7 Ethical Considerations

The study was conducted in accordance with the Declaration of Helsinki. Ethics approval was obtained from relevant national or institutional review boards and health research authorities in each centre. Data‐sharing agreements were established with all sites. All adolescents provided written informed consent or assent, as appropriate.

## 3 Results

### 3.1 Sample Characteristics

A total of 200 adolescents aged 12–17 years, mean age 14.5 (standard deviation (SD) = 1.6) years, 103 (51.5%) males, 97 (48.5%) females, were recruited from 10 countries (16 centres). Five ethnic groups were represented; almost half (48.5%) were of white ethnic origin. Most (79.5%) had at least one sibling, and 48.5% of adolescents attended school.

Sarcomas were the most common (*n* = 71, 36%) cancer type and the median time since diagnosis was 5.5 months (range 0–143). Forty percent of cancers were localised and 29% represented a relapse. Median time since the most recent treatment was 4.6 months (range 0–143); 191 (96%) adolescents were currently undergoing treatment and 175 (88%) treated with curative intent. Six (3%) had completed treatment within the past 12 months. Chemotherapy was the most common (*n* = 182, 91%) treatment modality. Seventy‐eight (39%) adolescents had an additional health condition, with anxiety (*n* = 15, 8%), depression (*n* = 10, 5%), and obesity (*n* = 12, 6%) the most common. Just over half of the adolescents (*n* = 104, 54%) rated themselves as fully active according to the ECOG Performance Scale. For a full overview of the characteristics of the adolescent participants, see Table 1.

**TABLE 1:** Socio‐demographic and clinical characteristics of adolescent participants *N* = 200.

Variable	
Participants recruited per country	Adolescents
Austria	14 (7.0%)
Belgium	27 (13.5%)
Croatia	3 (1.5%)
Denmark	19 (9.5%)
India	30 (15.0%)
Ireland	3 (1.5%)
Japan	20 (10.0%)
Jordan	31 (15.5%)
Spain	26 (13.0%)
UK	27 (13.5%)
Gender	
Female	97 (48.5%)
Male	103 (51.5%)
Age (years)	
Mean (standard deviation)	14.5 (1.6)
Ethnicity	
Arabic	35 (17.5%)
Asian	54 (27.0%)
Black	2 (1.0%)
Mixed	8 (4.0%)
White	97 (48.5%)
Not specified	4 (2.0%)
Education status	
Currently not receiving any education	13 (6.5%)
Discontinued education	2 (1.0%)
Educated at home	18 (9.0%)
Educated at hospital	8 (4.0%)
Educated at school (full‐time)	97 (48.5%)
Intermittent education	1 (0.5%)
Mixed home and school education	10 (5.0%)
Part‐time education at school	41 (20.5%)
Vacation	1 (0.5%)
Gap year	1 (0.5%)
Not specified	8 (4.0%)
Diagnosis	
Central nervous system	16 (8.0%)
Colon	1 (0.5)
Germ cell	7 (3.5%)
Kidney	2 (1.0%)
Leukaemia	50 (25.0%)
Liver	1 (0.5%)
Lymphoma	46 (23%)
Neuroblastoma	3 (1.5%)
Salivary gland	1 (0.5%)
Sarcoma	71 (35.5%)
Thyroid	1 (0.5%)
Unspecified	1 (0.5%)
Disease status	
Localised	80 (40.0%)
Metastatic	54 (27.0%)
Unspecified	13 (6.5%)
Relapse	
No	167 (83.5%)
Yes	29 (14.5%)
Unspecified	4 (2.0%)
Time since diagnosis (months)	
Median	5.5
Range	0–143
Unspecified	5 (2.5%)
Treatment status	
Currently on treatment	191 (95.5%)
Supportive/palliative care	3 (1.5%)
Treatment completed within the past year	6 (3.0%)
Treatment intent	
Curative	175 (87.5%)
Palliative	15 (7.5%)
Unspecified	10 (5.0%)
Time since treatment start (months)	
Median	4.6
Range	0–143
Unspecified	12 (6.0%)
Treatment type^a^	
Chemotherapy	182 (91.0%)
Hormonal	3 (1.5%)
Immunotherapy	13 (6.5%)
Radiotherapy	32 (16.0%)
Targeted therapy	14 (7.0%)
Co‐morbidities	
None	122 (61.0%)
One or more	78 (39.0%)
ECOG performance status	
0 (Fully active)	104 (52.0%)
1 (Restricted in physical strenuous activity)	63 (31.5%)
2 (Unable to carry out work activities)	19 (9.5%)
3 (Limited self‐care)	7 (3.5%)
Missing	7 (3.5%)

^a^ Total exceeds 200 because more than one treatment type recorded for participants.

### 3.2 Acceptability of the QLQ‐C30

Adolescents took on average 12 min to complete the QLQ‐C30 (mean = 12.14, SD = 9.01, range 1–40 min). Assistance was required by 42 (21%) adolescents to read out or clarify questions. The question on financial difficulties was identified as confusing by 20 (10%) adolescents and left unanswered by nine (5%) adolescents. Other questions which were identified as not age‐appropriate include carrying a heavy suitcase and reading a newspaper. The question asking respondents to consider whether cancer and its treatment have affected their family life was identified as difficult to answer by 12 (6%) adolescents. Questions which included words or phrases which they did not understand, or which were described as ‘medical‐type’ questions were identified as those asking about feeling tense (*n* = 11, 6%), irritable (*n* = 9, 5%), shortness of breath (*n* = 8, 4%), nauseated (*n* = 5, 3%), constipation (*n* = 4, 2%) and vomiting (*n* = 4, 2%).

Most participants (*n* = 192, 96%) did not find any of the QLQ‐C30 questions upsetting or inappropriate and for those who found at least one question upsetting this was due to not wanting to be reminded of their experiences. Twenty‐four adolescents (12%) recommended at least one question in need of re‐wording to add specificity (e.g., general quality of life question), clarity (e.g., definition of short or long walk) or to be ‘less personal’ (e.g., depression). Table 2 presents participants' feedback and recommendations. All questions were answered by more than 95% of participants.

**TABLE 2:** Feedback on the acceptability of QLQ‐C30 questions.

QLQ‐C30 item	No issues	Confusing/difficult to answer *n* (%)	Upsetting *n* (%)	Recommended for re‐wording *n* (%)	Missing *n* (%)	Comments
Carry heavy bag/suitcase	195 (97.5%)	4 (2%)	0 (0%)	1 (0.5%)	0 (0%)	Clarification for the meaning of heavy bag (*n* = 3). ‘I can carry things with my right side but not my left as this is weaker so I don't know how to answer.’ (DH02) Recommend re‐wording to remove reference to suitcase and replace with ‘teenage‐specific examples’ such as school bag (*n* = 2).
2 Long walk	191 (95.5%)	8 (4%)	0 (0%)	1 (0.5%)	1 (0.5%)	Clarification for the meaning of long walk (*n* = 5) How long is long? (SO09). ‘How many miles is this?’ (NU04). Environmental conditions (*n* = 1) ‘It depends on whether it's on a hill or not’ (JP13) Recommend re‐wording to include details about distance (*n* = 1)
3 Short walk	192 (96%)	7 (3.5%)	0 (0%)	1 (0.5%)	1 (0.5%)	Clarification for the meaning of short walk (*n* = 2). Dependent on the context: inside or outside (*n* = 1) Recommend re‐wording to include details about distance (*n* = 1)
4 Stay in bed/chair	195 (97.5%)	3 (1.5%)	0 (0%)	0 (0%)	2 (1%)	Variable (*n* = 1); ‘maybe you're not lying in bed all day, but sometimes you're lying on the couch’ (AT01) Dependent on the context (*n* = 1): ‘depends very much on whether he is at home or at the ward’ (AT12)
5 Help with self‐care	198 (99%)	2 (1%)	0 (0%)	0 (0%)	0 (0%)	Disagreement between parent and child (*n* = 1) Too many aspects to the question (*n* = 1)
6 Limited in work/daily activities	189 (94.5%)	10 (5%)	0 (0%)	1 (0.5%)	1 (0.5%)	Dependent on whether they are currently in hospital or at home: ‘I've been thinking about whether to include my daily life in the hospital or just consider my life at home.’ (JP08)
7 Limited in hobbies/leisure	194 (97%)	4 (2%)	0 (0%)	0 (0%)	2 (1%)	‘Almost every child can no longer pursue their hobbies’ (AT03) Recommend clarifying what is meant by leisure (*n* = 1)
8 Short of breath	192 (96%)	8 (4%)	0 (0%)	0 (0%)	4 (2%)	No comments shared
9 Pain	194 (97%)	4 (2%)	0 (0%)	0 (0%)	2 (1%)	Recommend clarifying type and location of pain (*n* = 4)
10 Need to rest	197 (98.5%)	2 (1%)	0 (0%)	1 (0.5%)	2 (1%)	Re‐wording to add clarity as to whether this is during the day (*n* = 1)
11 Trouble sleeping	198 (99%)	1 (0.5%)	0 (0%)	0 (0%)	1 (0.5%)	No comments shared
12 Weak	199 (99.5%)	0 (0%)	0 (0%)	0 (0%)	1 (0.5%)	No comments shared
13 Appetite loss	196 (98%)	2 (1%)	0 (0%)	0 (0%)	2 (1%)	Difficult to answer as appetite has never been good (*n* = 1).
14 Nauseated	192 (96%)	5 (2.5%)	1 (0.5%)	1 (0.5%)	1 (0.5%)	Recommend re‐wording to feeling sick (*n* = 1)
15 Vomited	194 (97%)	4 (2%)	1 (0.5%)	1 (0.5%)	0 (0%)	Difficult to quantify using the response scale (*n* = 1): ‘I have been sick once, is that a little bit?’ (AT06)
16 Constipated	194 (97%)	4 (2%)	1 (0.5%)	1 (0.5%)	0 (0%)	Uncomfortable to respond to (*n* = 1). Re‐word to have you had problems going to the toilet? (*n* = 1) ‘My Mum had to remind me what constipation was’ (BE14)
17 Diarrhoea	198 (99%)	0 (0%)	1 (0.5%)	1 (0.5%)	0 (0%)	Uncomfortable to respond to (*n* = 1) Re‐word to have you had problems going to the toilet? (*n* = 1)
18 Tired	199 (99.5%)	0 (0%)	0 (0%)	0 (0%)	1 (0.5%)	No comments shared
19 Pain interfering with daily activities	196 (98%)	3 (1.5%)	0 (0%)	0 (0%)	1 (0.5%)	Overlap with Q6 (limited in work/daily activities) (*n* = 1) Lack of clarity over the type of pain (physical or mental) (*n* = 1)
20 Difficulty concentrating	198 (99%)	1 (0.5%)	0 (0%)	1 (0.5%)	0 (0%)	Non‐age‐appropriate example used (*n* = 1); ‘I don't think people my age would read a newspaper’ (LD01). Re‐word to refer to concentrating while studying (*n* = 1)
21 Tense	185 (92.5%)	11 (5.5%)	2 (1%)	3 (1.5%)	0 (0%)	Overlap with Q22 (worry) (*n* = 1)
22 Worry	188 (94%)	7 (3.5%)	2 (1%)	1 (0.5%)	2 (1%)	Lack of clarity over the cause of worries (*n* = 1)
23 Irritable	189 (94.5%)	9 (4.5%)	2 (1%)	4 (2%)	1 (0.5%)	No comments shared
24 Depressed	194 (97%)	2 (1%)	2 (1%)	2 (1%)	0 (0%)	Recommend using the word sad instead of depressed (*n* = 1) ‘Depressed is a big word, very personal, I would prefer a more general question as it would help people feel more comfortable talking about this’ (SO03)
25 Difficulty remembering	198 (99%)	0 (0%)	0 (0%)	0 (0%)	2 (1%)	No comments shared
26 Family life interfered with	186 (93%)	12 (6%)	0 (0%)	0 (0%)	2 (1%)	Could not answer on behalf of family members and how they felt (*n* = 3) Recommend clarifying which members of the family referred to (*n* = 2)
27 Social activities interfered with	192 (96%)	7 (3.5%)	0 (0%)	1 (0.5%)	1 (0.5%)	Confusion over what social activities are referred to and whether these include time spent at school (*n* = 5)
28 Financial difficulties	171 (85.5%)	20 (10%)	2 (1%)	2 (1%)	9 (4.5%)	‘Question 28 is weird to young people. Because they live at home and don't have economic problems at all’ (DK02). Recommend re‐wording to be more age appropriate such as saving up money from holiday jobs (*n* = 1)
29 Overall health	189 (94.5%)	5 (2.5%)	0 (0%)	0 (0%)	6 (3%)	Response options (1–7) are too general (*n* = 2)
30 Overall quality of life	183 (91.5%)	13 (6.5%)	0 (0%)	2 (1%)	6 (3%)	Too general (*n* = 2): ‘I'm not sure what makes the quality of life good’

### 3.3 Content Validity

Most questions were recognised to some extent (i.e., ‘A little’ or more) by at least 30% (*n* = 60) adolescents (Table 3). Questions with the highest prevalence include tiredness (*n* = 163, 82%), needing to rest (*n* = 143, 72%) and difficulties taking a long walk (*n* = 140, 70%). Three questions, namely help with self‐care, vomiting and financial difficulties fell below the prevalence (30%) and mean (1.5) thresholds. The mean score for the questions about taking a short walk and diarrhoea also fell below 1.5 indicating low incidence.

**TABLE 3:** Performance of QLQ‐C30 questions: Adolescent participant responses to individual questions 1–28 and questions highlighted as irrelevant.

QLQ‐C30 item	Not at all *n* (%)	A little *n* (%)	Quite a bit *n* (%)	Very much *n* (%)	Prevalence ratio	Mean	Irrelevant *n* (%)
Carry heavy bag/suitcase	66 (33.0%)	83 (41.5%)	33 (16.5%)	18 (9.0%)	67.0%	2.0	1 (0.5%)
2 Long walk	59 (29.5%)	83 (41.5%)	34 (17.0%)	23 (11.5%)	70.0%	2.1	0 (0%)
3 Short walk	137 (68.5%)	44 (22.0%)	11 (5.5%)	7 (3.5)	31.0%	1.4	0 (0%)
4 Stay in bed/chair	103 (51.5%)	47 (23.5%)	30 (15.0%)	18 (9.0%)	47.5%	1.8	2 (1%)
5 Help with self‐care	154 (77.0%)	32 (16.0%)	8 (4.0%)	6 (3.0%)	23.0%	1.3	1 (0.5%)
6 Limited in work/daily activities	81 (40.5%)	67 (33.5%)	32 (16.0%)	19 (9.5%)	59.0%	1.9	2 (1%)
7 Limited in hobbies/leisure	76 (38.0%)	56 (28.0%)	35 (17.5)	31 (15.5%)	61.0%	2.1	2 (1%)
8 Short of breath	128 (64.0%)	48 (24.0%)	14 (7.0%)	6 (3.0%)	34.0%	1.5	1 (0.5%)
9 Pain	83 (41.5%)	70 (35.5%)	32 (16.0%)	13 (6.5%)	57.5%	1.9	0 (0%)
10 Need to rest	55 (27.5%)	85 (42.5%)	45 (22.5%)	13 (6.5%)	71.5%	2.1	1 (0.5%)
11 Trouble sleeping	104 (52.0%)	49 (24.5%)	31 (15.5%)	15 (7.5%)	47.5%	1.8	0 (0%)
12 Weak	59 (29.5%)	78 (39.0%)	43 (21.5%)	19 (9.5%)	70.0%	2.1	0 (0%)
13 Appetite loss	92 (46.0%)	56 (28.0%)	33 (16.5%)	17 (8.5%)	53.0%	1.9	0 (0%)
14 Nauseated	90 (45.0%)	69 (34.5%)	19 (9.5%)	21 (10.5%)	54.5%	1.9	2 (1%)
15 Vomited	147 (73.5%)	31 (15.5%)	10 (5.0%)	12 (6.0%)	26.5%	1.4	0 (0%)
16 Constipated	124 (62.0%)	44 (22.0%)	23 (11.5%)	9 (4.5%)	38.0%	1.6	2 (1%)
17 Diarrhoea	147 (73.5%)	33 (16.5%)	13 (6.5%)	7 (3.5%)	26.5%	1.4	1 (0.5%)
18 Tired	36 (18.0%)	96 (48.0%)	46 (23.0%)	21 (10.5%)	81.5%	2.3	0 (0%)
19 Pain interfering with daily activities	119 (59.5%)	55 (27.5%)	15 (7.5%)	10 (5.0%)	40.0%	1.6	0 (0%)
20 Difficulty concentrating	128 (64.0%)	49 (24.5%)	19 (9.5%)	4 (2.0%)	36.0%	1.5	2 (1%)
21 Tense	111 (55.5%)	62 (31.0%)	19 (6.5%)	8 (4.0%)	44.5%	1.6	2 (1%)
22 Worry	93 (46.5%)	67 (33.5%)	29 (14.5%)	9 (4.5%)	53.0%	1.8	2 (1%)
23 Irritable	85 (42.5%)	66 (33.0%)	32 (16.0%)	16 (8.0%)	57.0%	1.9	1 (0.5%)
24 Depressed	122 (61.0%)	49 (24.5%)	17 (8.5%)	12 (6.0%)	39.0%	1.6	2 (1%)
25 Difficulty remembering	130 (65.0%)	47 (23.5%)	14 (7.0%)	7 (3.5%)	34.0%	1.5	1 (0.5%)
26 Family life interfered with	96 (48.0%)	52 (26.0%)	30 (15.0%)	20 (10.0%)	51.0%	1.9	0 (0%)
27 Social activities interfered with	68 (34.0%)	53 (26.5%)	46 (23.0%)	32 (16.0%)	65.5%	2.2	1 (0.5%)
28 Financial difficulties	134 (67.0%)	40 (20.0%)	10 (5.0%)	7 (3.5%)	28.5%	1.4	28 (14%)

*Note:* Shaded cells correspond to cases where the thresholds have not been met in terms of relevance.

Forty‐two participants (21%) identified at least one irrelevant question (Table 3) with the question about financial difficulties the most frequently nominated (*n* = 28, 14%) due to their age: ‘question 28 is weird to young people because they live at home and don't have economic problems at all’. The example given for the question about concentration (reading a newspaper) was also queried for its age‐appropriateness. Some symptom questions, for example, nausea, constipation, diarrhoea and shortness of breath were marked as irrelevant as they were not common side‐effects of their type of cancer and treatment.

Fifty (25%) adolescents nominated at least one omission or area which needed further questioning, especially in relation to school life (*n* = 12, 6%) and friendships (*n* = 10, 5%).

Questions on loneliness and missing out on life and feeling different and ‘separate’ from peers were proposed. Boredom and lack of motivation to pursue activities were also suggested topics for questioning. A more in‐depth exploration of the emotional impact was recommended by nine adolescents: ‘Because I had so much psychological and emotional problems after each cycle of chemotherapy, I was surprised that the questionnaires didn't ask more on that.’ Three participants recommended questions on the impact on romantic relationships. Diet‐related questions and travel problems were suggested as omissions by five and three participants respectively.

### 3.4 Construct Validity

Evaluation of the eight‐factor structure of the QLQ‐C30 indicated an acceptable to good model fit across multiple indices (see Appendix S1). All questions demonstrated good to very strong standardised loadings on their respective subscales, supporting the internal coherence of the eight‐factor structure when used with adolescents (see Table 4).

**TABLE 4:** CFA standardised loadings by subscale and internal consistency.

Subscale	Questions	Range of loadings	Interpretation	Cronbach's alpha	Interpretation
Physical Functioning	Q1–Q5	0.60–0.77	Good—Strong	0.79	Acceptable
Role Functioning	Q6–Q7	0.76–0.80	Strong – Very Strong	0.78	Acceptable
Emotional Functioning	Q21–Q24	0.68–0.83	Good—Very Strong	0.84	Good
Cognitive Functioning	Q20, Q25	0.61–0.76	Good—Strong	0.70	Acceptable
Social Functioning	Q26, Q27	0.62–0.86	Good—Very Strong	0.70	Acceptable
Fatigue	Q10, Q12, Q18	0.67–0.70	Good—Strong	0.72	Acceptable
Nausea/Vomiting	Q14, Q15	0.66–0.97	Good—Very Strong	0.78	Acceptable
Pain	Q9, Q19	0.78–0.83	Strong – Very Strong	0.79	Acceptable

Internal reliability analysis further confirmed the subscale structure with acceptable‐good internal reliability of all subscales of the QLQ‐C30 with Cronbach's alpha values ranging from 0.70 to 0.84 (see Table 4).

### 3.5 Convergent and Discriminant Validity

Spearman correlations between conceptually similar subscales of the QLQ‐C30 and the PedsQL Cancer Teen provided at least moderate support of convergent validity (see Table 5) with all correlations ≥ 0.30. Lower correlations (< 0.30) between distinct subscales provide evidence for discriminant validity.

**TABLE 5:** Spearman correlations between conceptually similar and different QLQ‐C30 and PedsQL subscales.

QLQ‐C30 subscale	PedsQL subscale	Spearman correlation	Interpretation
Conceptually related subscales			
Pain	Pain_Hurt	0.60	Moderate–Strong, as expected
Nausea/Vomiting	Nausea	0.46	Moderate, supports convergent validity
Emotional Functioning	Worry	0.42	Moderate correlation, conceptually close
Emotional Functioning	Physical_Appearance	0.39	Somewhat related (body image ↔ emotions), moderate
Emotional Functioning	Procedural Anxiety	0.31	Low–Moderate, but still directionally correct
Emotional Functioning	Treatment Anxiety	0.36	Low–Moderate, supports convergent validity
Cognitive Functioning	Cognitive_Problems	0.45	Moderate, supports convergent validity
Social Functioning	Communication	0.30	Low–Moderate, but still directionally correct
Conceptually distinct subscales			
Pain	Physical_Appearance	0.13	Low association, supports discriminant validity
Nausea/Vomiting	Physical_Appearance	0.00	Low association, supports discriminant validity
Cognitive Functioning	Pain_Hurt	0.27	Low association, supports discriminant validity

### 3.6 Known‐Group Validity

For treatment intent, differences between groups were generally negligible to small. Small effect sizes were observed for emotional functioning (*r* = 0.223), cognitive functioning (*r* = 0.167), nausea/vomiting (*r* = 0.140), financial difficulties (*r* = 0.149) and global health status/QoL (*r* = 0.166), with patients in the palliative group generally reporting poorer outcomes. All remaining subscales demonstrated negligible to very small effects (*r* < 0.10), indicating limited discrimination between curative and palliative groups for most domains.

In contrast, performance status showed clearer group differences. Moderate effect sizes were observed for role functioning (*r* = 0.356), physical functioning (*r* = 0.342) and fatigue (*r* = 0.337), while small‐to‐moderate effects were found for social functioning (*r* = 0.275), pain (*r* = 0.256), cognitive functioning (*r* = 0.207) and diarrhoea (*r* = 0.111). These differences indicate worse functioning and symptom burden among patients who were not fully active (ECOG 1–3). Other subscales, including emotional functioning (*r* = 0.021), nausea/vomiting (*r* = 0.074), dyspnoea (*r* = 0.009), sleep disturbance (*r* = 0.095) and appetite loss (*r* = 0.062), showed negligible to small effects (Table 6).

**TABLE 6:** Known‐group validity of the QLQ‐C30.

Subscale/question	Median (IQR)	Median (IQR)	Effect size (*r*)	*p*
Group comparison	Curative	Palliative		
Treatment intent				
Physical functioning	80.0 (26.7)	80.0 (20.0)	0.002	0.982
Role functioning	66.7 (50.0)	100.0 (50.0)	0.081	0.258
Emotional functioning	83.3 (25.0)	100.0 (9.7)	0.223	0.002
Cognitive functioning	83.3 (33.3)	100.0 (0.0)	0.167	0.013
Social functioning	66.7 (50.0)	83.3 (50.0)	0.074	0.303
Fatigue	66.7 (33.3)	55.6 (11.1)	0.013	0.855
Nausea_Vomiting	83.3 (33.3)	100.0 (16.7)	0.140	0.043
Pain	83.3 (41.7)	83.3 (33.3)	0.033	0.640
Dyspnoea	100.0 (33.3)	100.0 (33.3)	0.044	0.480
Sleep disturbance	66.7 (33.3)	100.0 (33.3)	0.085	0.206
Appetite loss	66.7 (66.7)	66.7 (33.3)	0.018	0.795
Constipation	100.0 (33.3)	100.0 (50.0)	0.047	0.454
Diarrhoea	100.0 (33.3)	100.0 (16.7)	0.012	0.837
Financial difficulties	100.0 (33.3)	100.0 (0.0)	0.149	0.012
Health status/QoL	66.7 (41.7)	83.3 (37.5)	0.166	0.024

Group comparison	Fully active (ECOG = 0)	Not fully active (ECOG 1–3)		
Performance status				
Physical functioning	86.7 (20.0)	73.3 (26.7)	0.342	< 0.001
Role functioning	83.3 (33.3)	66.7 (50.0)	0.356	< 0.001
Emotional functioning	83.3 (35.4)	83.3 (25.0)	0.021	0.770
Cognitive functioning	100.0 (16.7)	83.3 (33.3)	0.207	0.002
Social functioning	83.3 (50.0)	66.7 (50.0)	0.275	< 0.001
Fatigue	66.7 (33.3)	55.6 (33.3)	0.337	< 0.001
Nausea_Vomiting	83.3 (33.3)	83.3 (33.3)	0.074	0.277
Pain	83.3 (33.3)	66.7 (33.3)	0.256	< 0.001
Dyspnoea	100.0 (33.3)	100.0 (33.3)	0.009	0.884
Sleep disturbance	100.0 (33.3)	66.7 (66.7)	0.095	0.152
Appetite loss	66.7 (33.3)	66.7 (66.7)	0.062	0.362
Constipation	100.0 (33.3)	100.0 (33.3)	0.040	0.519
Diarrhoea	100.0 (0.0)	100.0 (33.3)	0.111	0.047
Financial difficulties	100.0 (33.3)	100.0 (33.3)	0.029	0.628
Health status/QoL	75.0 (33.3)	66.7 (31.2)	0.069	0.340

### 3.7 Preferences

#### 3.7.1 Questionnaire Type

Over half (*n* = 117, 59%) adolescents preferred the PedsQL, 29% (*n* = 58) preferred the QLQ‐C30, 8% (*n* = 15) had no preference and 5% (*n* = 10) did not specify. Reasons for preferring the PedsQL included the questionnaire format (*n* = 33, 17%), specificity and level of detail (*n* = 26, 13%), comprehensiveness (*n* = 10, 5%) and relevance to a younger age group (*n* = 14, 7%). A similar set of reasons for favouring the QLQ‐C30 was also reported including ease of completion due to shorter, clearer sentences (*n* = 25, 13%), better layout and response options (*n* = 8, 4%), more comprehensive (*n* = 9, 5%), shorter length (*n* = 6, 3%) and greater relevance to their age group (*n* = 10, 5%).

#### 3.7.2 Mode of Delivery

More adolescents (*n* = 113, 57%) preferred completing the questionnaire via paper compared with electronically (*n* = 49, 25%) while 38 (19%) had no preference. No trends for preference were observed across countries. Reasons for favouring paper completion include familiarity and completion in their own time. There was a concern that technology and internet connections could be unreliable. Conversely, some adolescents described the direct electronic submission of answers as easier and not prone to being lost. Regardless of preferred mode of delivery, seven adolescents recommended having someone with them while completing the questions to assist with any queries.

## 4 Discussion

This study is the first of its kind to evaluate the acceptability and validity of an adult HRQoL measure, the QLQ‐C30, with a large group of adolescents with cancer aged 12–17 years from 10 countries worldwide. Although the QLQ‐C30 was developed for and tested with adults ≥ 18 years, with a mean age of 63 years in the original development and validation work [7], our study provides evidence that its psychometric properties remain robust when applied to younger patients, even superior for some tests; for example, scale reliability with Cronbach's alpha of role functioning 0.52 in the original testing compared with 0.78 in the current study.

The current study confirmed the structure of the eight QLQ‐C30 symptom and functioning subscales and internal reliability of their questions. The relationship between the QLQ‐C30 and PedsQL subscales was as expected based on their conceptual relevance, further supporting its validity.

Further support for the psychometric properties of the QLQ‐C30 was observed in its ability to discriminate between groups defined by performance status, with small‐to‐moderate effect sizes across physical, role, and fatigue domains. In contrast, differences between treatment intent groups were generally small. This pattern is consistent with previous research indicating that the QLQ‐C30 more readily discriminates between groups defined by proximal indicators of functional status, such as ECOG Performance Status, than by broader clinical categories that may be more heterogeneous and less directly related to current HRQoL [45]. In adolescent populations, the distinction between curative and palliative treatment may not correspond closely to symptom burden at a single time point, which may attenuate observed differences. Taken together, these findings suggest that, in cross‐sectional analyses, the QLQ‐C30 demonstrates stronger known‐group validity for functional status than for treatment intent.

Further support for the psychometric properties of the QLQ‐C30 was offered in terms of its ability to discriminate between groups of adolescents based on their performance status and treatment intent.

Feedback from adolescents suggested that the QLQ‐C30 was largely acceptable, easy to complete, and captured issues which were meaningful for their age group, in particular relating to physical functioning and fatigue. A similar pattern of question relevance was reported in the recent content validation and acceptability of the QLQ‐C30 with adults [11]. In the current study, most issues addressed were recognised to some extent by at least 30% adolescents and compliance was high, with all questions answered by more than 95% adolescents. Further evidence supporting acceptability of the QLQ‐C30 is the mean completion time of 12 min which is comparable to data gathered from adults [46]. The presence of a researcher to assist participants could explain the good performance of questions, reflected in the high compliance across all questions.

Despite this study providing widespread support for the QLQ‐C30, there were recommendations to improve the wording of specific questions and provide age‐appropriate examples. While this feedback is valuable and underscores the importance of developmental appropriateness, any modification to the wording of the QLQ‐C30 would require careful evaluation. Even minor changes to item phrasing may alter the underlying construct being measured and would necessitate formal psychometric revalidation. Moreover, adapting the questionnaire for adolescents could limit comparability with existing adult datasets and undermine one of the key strengths of the QLQ‐C30, namely its widespread use across age groups and clinical settings. As such, while the suggested wording refinements may inform future instrument development or adaptation, further research is needed to determine whether such modifications improve measurement properties without compromising validity or comparability.

The preference for the PedsQL among adolescents is not unexpected and likely reflects its age‐appropriate design, including language, content, and framing tailored to younger populations. In contrast, the QLQ‐C30 was developed for adults and may include concepts or wording that are less immediately resonant for younger respondents. Nevertheless, a third of adolescents preferred the QLQ‐C30, describing it as user‐friendly and concise.

A quarter of adolescents highlighted aspects of their experience which had been overlooked by the QLQ‐C30, such as school engagement, peer interaction, boredom and autonomy which are only broadly covered by the questions on limitations to work or daily activities and interference with social activities. These missing issues reflect critical developmental domains that are central to adolescent well‐being and underscore the necessity of incorporating adolescents' psychosocial environment, developmental stage and priorities into HRQoL assessments for this age group.

It is not surprising that the QLQ‐C30 omits some aspects of adolescents' experiences with cancer, given that this generic measure was designed for adults with cancer who have a higher cancer incidence and it is well recognised that the cancer experience of adolescents is distinct [47, 48]. Interestingly, several of these HRQoL issues were also identified as missing from the PedsQL. The EORTC measurement model [37] recommends the use of the QLQ‐C30 alongside questions specific to a particular area of interest (disease, symptom or age group) to enhance its sensitivity for the target population. The current study supports the use of the QLQ‐C30 alongside the EORTC measure specific to adolescents and young adults, aged 14–39 years, the EORTC QLQ‐AYA30 (QLQ‐AYA30) [49]. In addition, an EORTC measure for children < 14 years is currently in development [50].

Our study revealed an unexpected preference for paper‐based questionnaires which underscores the need for careful consideration as we move towards a greater emphasis on remote health assessments. Flexible options should be offered for questionnaire completion to support accessibility, including the presence of someone to assist if needed.

### 4.1 Strength and Limitations

Although we recruited a diverse sample of adolescents across 10 countries, there was a bias towards participants of white ethnicity. The cross‐sectional study design and sample size of 200 did not permit more extensive validity and reliability testing, such as test–retest reliability, responsiveness to change, and item response theory analysis. Adolescents were not consecutively recruited, and details regarding non‐responders were not collected. In addition, the presence of a researcher to support the questionnaire delivery might have contributed favourably to the performance of the QLQ‐C30, and positive appraisals regarding the QLQ‐C30 might have been encouraged by the study's affiliation to the EORTC. Notwithstanding these limitations, this study has several important strengths. The international, multi‐centre design enabled recruitment of adolescents across a range of healthcare settings, supporting the generalisability of the findings. The study also addresses a relative gap in the literature by evaluating the performance of the QLQ‐C30 in an adolescent oncology population. The inclusion of the PedsQL as a comparator instrument strengthened the interpretation of findings, while the incorporation of qualitative feedback provided additional insight into questionnaire acceptability and relevance from the adolescent perspective.

### 4.2 Recommendations

The findings from our cross‐cultural validation study support the use of the QLQ‐C30 among adolescents aged 12–17 years, establishing it as a core measure of choice for assessing outcomes in clinical trials, research and clinical practice for this age group. To enhance measurement precision and capture AYA‐specific concerns, the QLQ‐AYA30 is recommended as a complementary instrument. Given its brevity, global availability and strong psychometric performance, the QLQ‐C30 can be readily integrated into trial protocols for adolescents. Research to further evaluate the psychometric properties of the QLQ‐C30 in a larger sample of adolescents would be insightful alongside a consideration of adolescents' preferences for PRO completion to ensure that assessments are meaningful and not burdensome.

## 5 Conclusion

The QLQ‐C30 is recommended as a core HRQoL measure for adolescents in clinical trials, providing a standardised framework for assessment. Its use enables meaningful comparisons across age groups and facilitates longitudinal data collection. Furthermore, this validation study helps to address an important barrier to the inclusion of adolescents in clinical trials and supports regulatory expectations for the use of PROs in this population.

## Author Contributions

**Haneen Abaza:** writing – review and editing. **David Riedl:** writing – review and editing. **Lisa Lyngsie Hjalgrim:** writing – review and editing. **Andrea Artigas Ramiro:** writing – review and editing. **Amal Al‐Omari:** writing – review and editing. **Chiara Besani:** writing – review and editing. **Betty Illatopa:** writing – review and editing. **Carlota Aguilera Ordoñez:** writing – review and editing. **Samantha C. Sodergren:** conceptualization, methodology, project administration, data curation, writing – original draft, writing – review and editing. **Lucas Moreno:** writing – review and editing. **Manjunath Nookala Krishnamurthy:** writing – review and editing. **Jamal Hossain:** formal analysis, writing – review and editing. **Anna Salo:** writing – review and editing. **Rebecca Mottram:** writing – review and editing. **Jaques Van Heerden:** writing – review and editing. **Stephanie O'Toole:** writing – review and editing. **Caroline De Schepper:** writing – review and editing. **Hiroto Ishiki:** writing – review and editing. **Marta Pérez‐Campdepadros:** writing – review and editing. **Jelena Roganovic:** writing – review and editing. **Mark P. Tighe:** writing – review and editing. **Anne‐Sophie Darlington:** writing – review and editing.

## Funding

This research was funded by the European Organization for Research and Treatment of Cancer (EORTC) Quality of Life Group, grant numbers [002‐2018]. The EORTC QLG business model involves license fees for commercial use of their instruments. Academic use of EORTC instruments is free of charge.

## Disclosure

The funder reviewed and approved the study proposal but had no direct influence on the design and conduct of the study; collection, management, analysis and interpretation of the data. The final manuscript was submitted to and approved by the Executive Committee of EORTC QLG, and the manuscript is published on behalf of the EORTC QLG. Professor Anne‐Sophie Darlington as Principal Investigator had full access to all the data in the study and takes responsibility for the integrity of the data and the accuracy of the data analysis.

## Ethics Statement

This study was conducted in accordance with the principles of the Declaration of Helsinki. Ethical approval was obtained from the London‐Bloomsbury Research Ethics Committee (Reference 20/PR/0170) on 28 September 2020.

## Consent

Written informed consent was obtained from all participants prior to enrolment. For participants below the age of legal consent in the recruiting country, assent was obtained from the participant and written informed consent was obtained from a parent or legal guardian. All methods were carried out in accordance with relevant guidelines and regulations.

## Conflicts of Interest

The authors declare no conflicts of interest.

## Supporting information


**Appendix S1:** cam471952‐sup‐0001‐AppendixS1.zip.

## Data Availability

Data are shared according to the EORTC data sharing process and accessible via the EORTC Quality of Life Group Repository https://qol.eortc.org/projectqol/data‐repository/.
