# Supplementary material for: Assessing the Validity and Acceptability of an Adult Quality of Life Questionnaire, the EORTC QLQ‐C30, for Adolescents With Cancer
Source: Cancer Med. 2026 May 21;15(5):e71952. doi: 10.1002/cam4.71952 (PMC13239106; doi:10.1002/cam4.71952)
Supplement: Supplementary file 1 — Appendix S1: cam471952‐sup‐0001‐AppendixS1.zip. [file CAM4-15-e71952-s001.zip › cam471952-sup-0001-Supinfo1@Summary_statements_for_Table_of_Contents.docx]

Summary statements for Table of Contents

- This international study demonstrates that the EORTC QLQ-C30 is acceptable, reliable, and valid for adolescents aged 12–17 years with cancer, supporting its use in clinical trials with younger patients.
- By confirming the robust psychometric performance and feasibility across cultures, this work enables a lifecourse based patient-reported outcome assessment as trial age-eligibility is lowered.
