# Supplementary material for: Assessing the Validity and Acceptability of an Adult Quality of Life Questionnaire, the EORTC QLQ‐C30, for Adolescents With Cancer
Source: Cancer Med. 2026 May 21;15(5):e71952. doi: 10.1002/cam4.71952 (PMC13239106; doi:10.1002/cam4.71952)
Supplement: Supplementary file 1 — Appendix S1: cam471952‐sup‐0001‐AppendixS1.zip. [file CAM4-15-e71952-s001.zip › cam471952-sup-0002-TableS1@Supplementary_material_etable1.docx]

Supplementary material

Table S1. Model Fit Indices from Confirmatory Factor Analysis

| Fit Index | Value | Interpretation |
| --- | --- | --- |
| Chi-Square (χ²) test  p-value | 0.061 | Insignificant – suggests the model fits well |
| Comparative Fit Index | 0.94 | Good fit (≥ 0.90 acceptable, ≥ 0.95 excellent) |
| Tucker Lewis Index | 0.924 | Good fit (≥ 0.90 acceptable) |
| Root Mean Square Error or Approximation | 0.053* | Good fit (< 0.06 good, < 0.08 acceptable) |
| Standardised Root Mean Residual | 0.056 | Good fit (< 0.08 acceptable, < 0.05 excellent) |

*90% Confidence Interval for RMSEA [0.035, 0.071]
